# Beyond prognosis: Identity, meaning, and existential uncertainty after severe brain injury

**DOI:** 10.1017/S1478951526103216

**Published:** 2026-08-10

**Authors:** Eric Wagner da Silva Picon-Trevisanuto, Felipe de Lacerda-Pereira, Miguel Augusto-rodrigues, Rafela Melo Macedo, Júlia Drummond de Camargo, João Carlos Geber-Junior

**Affiliations:** 1 Hospital das Clínicas, Faculdade de Medicina, Universidade de São Paulo, São Paulo, Brazil; 2 Department of Internal Medicine, Hospital de Base do Distrito Federal, Brasília, Brazil; 3 Ribeirão Preto Medical School, University of São Paulo, Ribeirão Preto, Brazil; 4 Palliative Care Team, Hospital Samaritano de São Paulo, São Paulo, Brazil; 5 Instituto do Câncer do Estado de São Paulo (ICESP), Hospital das Clínicas da Faculdade de Medicina da Universidade de São Paulo, São Paulo, Brazil; 6 Universidade de Sao Paulo Faculdade de Medicina, Brazil; 7 Palliative Care Support Team, Hospital Samaritano de São Paulo, Brazil

Modern medicine is built upon prediction. Clinicians estimate risks, forecast outcomes, and construct probabilities intended to guide decisions about treatment and care. In many circumstances, these predictions provide a foundation for clinical reasoning and shared decision-making. Yet severe neurological illness exposes a distinctive limitation of this enterprise. Some of the questions that matter most to patients and families cannot be answered through prediction alone.

Advances in neuroprognostication have substantially improved clinicians’ ability to estimate survival and functional recovery after severe brain injury. Neuroimaging, electrophysiological monitoring, biomarkers, and standardized prognostic frameworks have increased the precision with which outcomes can be anticipated (Faiver and Steinberg 2025). Recent observations of cognitive motor dissociation have further refined understanding of consciousness by demonstrating that some patients who appear entirely unresponsive may retain covert cognitive function undetectable through bedside examination alone (Bodien et al. 2024). These developments represent genuine progress. They have improved the accuracy of prognostic assessment and expanded the boundaries of what medicine can know.

Yet greater predictive precision has not resolved a more fundamental problem. Prognosis may estimate survival, disability, and functional recovery with increasing sophistication, but it remains far less capable of determining what those outcomes will ultimately mean to the person who experiences them. When a devastating brain injury occurs, families often ask questions that appear medical but are ultimately existential. Will she wake up? Will he recognize us again? Will life still be worth living? These questions concern more than biological outcomes. They concern identity, meaning, and the future significance of a life that has not yet unfolded. The central limitation of prognosis is not merely uncertainty about outcomes, but uncertainty about the meaning those outcomes may ultimately acquire.

This challenge acquires particular ethical significance because severe neurological illness frequently removes the patient’s voice precisely when that voice is most needed. Decisions regarding life-sustaining treatment, rehabilitation, and goals of care are often made in the absence of the person most affected by them. The resulting dilemma extends beyond the loss of decisional capacity. It reflects the ethical challenge of caring for persons whose vulnerability may exceed the explanatory reach of autonomy alone (Geber-Junior 2025). It reflects uncertainty about personhood itself. The relevant question is not merely what the patient would have wanted before injury, but whether those preferences remain fully applicable to a future self whose capacities, relationships, and sources of meaning may be transformed.

As previously argued, autonomy under conditions of vulnerability may be better understood as a relational and evolving process than as the simple preservation of prior preferences (Geber-Junior and Forte 2025). Severe neurological illness complicates this further because the self to whom decisions are directed remains partially unknown. Under such circumstances, autonomy may be better understood not as a fixed capacity but as a fragile and relational achievement that emerges through dialogue, interpretation, and sustained ethical attention (Geber-Junior 2026a). Surrogates are therefore asked to make decisions at the intersection of memory and uncertainty, attempting to remain faithful to a person whose future experience remains inaccessible to everyone involved (Shalowitz et al. 2006).

Shared decision-making seeks to bridge this uncertainty by grounding decisions in what is known about the patient’s life before illness. This approach assumes that previously expressed values, relationships, and life narratives provide meaningful guidance for decisions made in the present. Ricoeur’s concept of narrative identity suggests that personal identity is sustained not through permanence, but through continuity across time, relationships, and lived experience (Ricoeur 1992). Yet severe neurological injury challenges precisely this continuity. It may alter cognition, communication, identity, relationships, and lived experience. The future under discussion is not simply an outcome. It is a life.

This distinction matters because prognostic conversations often contain an implicit question that neither families nor clinicians can answer with confidence: would this future life still be meaningful? Beneath discussions of survival and disability lies a deeper concern about whether life under radically altered circumstances can still sustain dignity, purpose, connection, and value.

Evidence suggests that human beings are remarkably poor at making such judgments in advance. The disability paradox demonstrates that many individuals living with severe disability report levels of well-being that external observers would not anticipate (Albrecht and Devlieger 1999). Similarly, research on affective forecasting shows that people consistently overestimate the enduring burden of future suffering while underestimating their capacity for adaptation (Peters et al. 2014). What appears intolerable from the perspective of the present may be experienced very differently once it becomes part of lived reality. Human beings adapt. Values evolve. Lives are reinterpreted.

Response shift theory offers one explanation for this phenomenon. Individuals confronted with serious illness frequently recalibrate internal standards, reprioritize values, and redefine quality of life (Sprangers and Schwartz 1999). Adaptation is not merely resilience. It is a reconstruction of value, identity, and meaning. What patients value after serious illness may differ profoundly from what they imagined they would value before it. Consequently, prognostic estimates may describe future functional states while remaining unable to anticipate the significance those states will eventually acquire.

These observations reveal an important epistemological boundary within medicine. Prognostic models may estimate mortality and functional impairment, but they cannot reliably anticipate resilience, adaptation, or the significance that a future life may hold for the person who eventually lives it. Their strength lies in describing probabilities; their limitation lies in addressing meaning.

The ethical error arises when predictive knowledge is mistaken for existential knowledge.

Prognostic estimates often acquire an authority that exceeds their actual scope, particularly in moments of crisis when families seek clarity and clinicians feel compelled to provide it. Under such circumstances, prognostic estimates can cease to function merely as descriptions of possibility and begin to shape the decisions from which outcomes emerge. This dynamic is particularly important in severe neurological illness, where predictions of poor outcome may influence decisions to withdraw life-sustaining treatment, creating the possibility of self-fulfilling prophecy (Becker et al. 2001; Turgeon et al. 2011).

Recognizing this reality calls for prognostic humility. Such humility does not require therapeutic hesitation or avoidance of difficult conversations. Rather, it reflects an awareness of the boundaries between what medicine can estimate and what it cannot know. Clinicians can describe trajectories, but they cannot inhabit the future they are attempting to describe. This perspective aligns with contemporary discussions of prognostic humility following severe brain injury (Kreitzer et al. 2023).

For palliative care, this distinction carries an important implication. If prognosis cannot fully anticipate the significance that a future life may acquire, then uncertainty should not be regarded as an obstacle to care. It is one of the conditions that makes care necessary. Patients and families continue to require interpretation, continuity, and accompaniment even when prediction reaches its limits. As previously argued, uncertainty is not a failure of medicine but the terrain within which some of the most important work of care occurs (Geber-Junior and Forte 2025).

The limits of prediction do not diminish the importance of care. They redefine it.

When intervention reaches its limits, care does not disappear but undergoes a transformation of purpose. The clinical significance of non-intervention lies not in therapeutic withdrawal but in recognizing forms of care that remain possible despite the inability to modify disease trajectories. Severe neurological illness reminds us that some of the most important responsibilities of care emerge precisely when outcomes cannot be controlled. In such moments, remaining present is not what medicine does after its work is finished. It is part of the work itself. This understanding is consistent with an emerging view of palliative care as a clinical stance that precedes prognostic certainty and remains relevant despite it (Geber-Junior and Forte 2026).

Ultimately, the ethical challenge of neuroprognostication is not merely determining what is likely to happen. It is recognizing the limits of what can be known about the life that may emerge if survival occurs. Prognosis can estimate outcomes. It cannot predict meaning. This distinction suggests the existence of a second layer of uncertainty that remains irreducible to prognostic accuracy. Prognostic uncertainty concerns what is likely to happen. Existential uncertainty concerns what survival will ultimately mean to the person who lives it. The deepest uncertainty in severe neurological illness is not whether survival will occur, but what survival will ultimately come to mean.

The questions that matter most remain the ones that prediction cannot answer. Until then, the task of medicine is not to replace uncertainty with certainty, but to ensure that uncertainty is not faced alone.

## References

[ref1] Albrecht GL and Devlieger PJ (1999) The disability paradox: High quality of life against all odds. *Social Science & Medicine* 48(8), 977–988. doi:10.1016/S0277-9536(98)00411-0 10390038

[ref2] Becker KJ, Baxter AB, Cohen WA, et al. (2001) Withdrawal of support in intracerebral hemorrhage may lead to self-fulfilling prophecies. *Neurology* 56(6), 766–772. doi:10.1212/WNL.56.6.766 11274312

[ref3] Bodien YG, Allanson J, Cardone P, et al. (2024) Cognitive motor dissociation in disorders of consciousness. *New England Journal of Medicine* 391(7), 598–608. doi:10.1056/NEJMoa2400645. 39141852 PMC7617195

[ref4] Faiver L and Steinberg A (2025) Timing of neuroprognostication in the ICU. *Current Opinion in Critical Care* 31(2), 155. doi:10.1097/MCC.0000000000001241 39808443 PMC12581068

[ref5] Geber-Junior JC (2025) When autonomy fails: Ethics, philosophy, and the legal duty of palliative care—Reflections on end-of-life medicine in the 21st century. *Palliative and Supportive Care* 23, e200. doi:10.1017/S1478951525101028 41185425 PMC13166487

[ref6] Geber-Junior JC (2026a) Possible autonomy: Finitude, vulnerability, and the ethical duty of listening. *Palliative and Supportive Care* 24, e111. doi:10.1017/S1478951526102375 42003019 PMC13166440

[ref7] Geber-Junior JC (2026b) Beyond intervention: The clinical meaning of non-intervention in serious illness. *Palliative and Supportive Care* 24, e131. doi:10.1017/S1478951526102545 42077013 PMC13166255

[ref8] Geber-Junior JC and Forte DN (2025) The anatomy of connection: Rethinking communication beyond the script. *Palliative and Supportive Care* 23, e216. doi:10.1017/S1478951525101235 41431264 PMC13166344

[ref9] Geber-Junior JC and Forte DN (2026) Palliative care in emergency medicine: A perspective from a trainee. *Annals of Emergency Medicine*. doi:10.1016/j.annemergmed.2026.05.002 42313043

[ref10] Kreitzer N, Murtaugh B, Creutzfeldt C, et al. (2023) Prognostic humility and ethical dilemmas after severe brain injury: Summary, recommendations, and qualitative analysis of Curing Coma Campaign virtual event proceedings. *Frontiers in Human Neuroscience* 17, 1128656. doi:10.3389/fnhum.2023.1128656. 37063099 PMC10102639

[ref11] Peters SA, Laham SM, Pachter N, et al. (2014) The future in clinical genetics: Affective forecasting biases in patient and clinician decision making. *Clinical Genetics* 85(4), 312–317. doi:10.1111/cge.12255 23952534

[ref12] Ricoeur P (1992) *Oneself as Another*. Chicago: University of Chicago Press.

[ref13] Shalowitz DI, Garrett-mayer E and Wendler D (2006) The accuracy of surrogate decision makers: A systematic review. *Archives of Internal Medicine* 166(5), 493–497. doi:10.1001/archinte.166.5.493 16534034

[ref14] Sprangers MAG and Schwartz CE (1999) Integrating response shift into health-related quality of life research: A theoretical model. *Social Science & Medicine* 48(11), 1507–1515. doi:10.1016/S0277-9536(99)00045-3 10400253

[ref15] Turgeon AF, Lauzier F, Simard J-F, et al. (2011) Mortality associated with withdrawal of life-sustaining therapy for patients with severe traumatic brain injury: A Canadian multicentre cohort study. *Canadian Medical Association Journal* 183(14), 1581–1588. doi:10.1503/cmaj.101786. 21876014 PMC3185074

